# Cryopreservation and Resuscitation of Natural Aquatic Prokaryotic Communities

**DOI:** 10.3389/fmicb.2020.597653

**Published:** 2021-01-28

**Authors:** Angel Rain-Franco, Guilherme Pavan de Moraes, Sara Beier

**Affiliations:** ^1^UMR 7621 Laboratoire d’Océanographie Microbienne, Observatoire Océanologique de Banyuls-sur-Mer, Sorbonne Université, Banyuls-sur-Mer, France; ^2^Graduate Program in Ecology and Natural Resources (PPGERN), Laboratory of Phycology, Department of Botany, Universidade Federal de São Carlos, São Carlos, Brazil; ^3^Department of Biological Oceanography, Leibniz Institute for Baltic Sea Research Warnemünde, Rostock, Germany

**Keywords:** cryobiology, cryopreservation, culture, aquatic environments, complex microbial communities, microbial ecology, experimental microbiology, community composition

## Abstract

Experimental reproducibility in aquatic microbial ecology is critical to predict the dynamics of microbial communities. However, controlling the initial composition of naturally occurring microbial communities that will be used as the inoculum in experimental setups is challenging, because a proper method for the preservation of those communities is lacking. To provide a feasible method for preservation and resuscitation of natural aquatic prokaryote assemblages, we developed a cryopreservation procedure applied to natural aquatic prokaryotic communities. We studied the impact of inoculum size, processing time, and storage time on the success of resuscitation. We further assessed the effect of different growth media supplemented with dissolved organic matter (DOM) prepared from naturally occurring microorganisms on the recovery of the initially cryopreserved communities obtained from two sites that have contrasting trophic status and environmental heterogeneity. Our results demonstrated that the variability of the resuscitation process among replicates decreased with increasing inoculum size. The degree of similarity between initial and resuscitated communities was influenced by both the growth medium and origin of the community. We further demonstrated that depending on the inoculum source, 45–72% of the abundant species in the initially natural microbial communities could be detected as viable cells after cryopreservation. Processing time and long-term storage up to 12 months did not significantly influence the community composition after resuscitation. However, based on our results, we recommend keeping handling time to a minimum and ensure identical incubation conditions for repeated resuscitations from cryo-preserved aliquots at different time points. Given our results, we recommend cryopreservation as a promising tool to advance experimental research in the field of microbial ecology.

## Introduction

Experimental studies have played a central role in the field of microbial ecology over the past 55 years (Duarte et al., 1997; Shade et al., 2012) and have been critical to our comprehension of microbial dynamics and processes in aquatic ecosystems (Bell et al., 2009). Such studies are particularly valuable to reduce the complexity of natural systems by artificially controlling conditions while separately assessing the impact of selected parameters on community dynamics and functioning.

However, their confidence and reproducibility rely on maintaining comparable initial conditions, which include the identity and density of organisms in the starting community. Differences in community initial composition and inherent environmental variability may be propagated and amplified during the course of an experiment (Fukami, 2015), effectively affecting both the final community composition and functioning (Bloesch et al., 1988). Particularly, the option to perform temporally delayed follow-up experiments, for instance, with the aim of changing certain parameters of interest based on the outcome of an initial experiment, depends on the availability of standardized starting communities.

The assembly of bacterial strains into artificial communities to be used as starting communities in experimental setups is one option to circumvent these caveats and it has been applied in multiple studies to test ecological theory, such as the biodiversity-ecosystem functioning (BEF) relationships (e.g., Bell et al., 2005; Matias et al., 2013; García et al., 2018). Experimental ecological studies usually aim to elucidate generally valid ecological patterns that do not depend on the presence of particular species. Therefore, deviations in the species composition of experimental communities compared to naturally occurring communities are usually accepted. However, while experimental approaches based on artificial communities did, to our knowledge, maximally include 72 different species (Bell et al., 2005; Shakya et al., 2013; Singer et al., 2016; Yu et al., 2016; Cairns et al., 2018), natural microbial communities are usually highly diverse assemblages containing several hundreds of species (Ibarbalz et al., 2019; Salazar et al., 2019). Even though the reduced complexity of the artificial communities allows the elucidation of the main drivers behind community performance and stability (Großkopf and Soyer, 2014), it is unknown whether the complexity of natural microbial communities is accurately represented in this approach (Wolfe, 2018). For instance, it has been observed that community functioning is strongly linked to changing species diversity in low diversity mock communities, while BEF relationships are less predictable in highly diverse communities, which is typical for natural microbial communities (Bell et al., 2009).

To better link the findings from experiments performed on a limited number of strains with processes taking place in natural environments, experimental manipulation of naturally co-occurring microbes is a valuable tool (e.g., Bell et al., 2005). However, while the control over the composition and density of starting communities of simple consortia comprised of few species is rather easy, controlling the initial composition of complex, naturally occurring microbial communities is challenging.

The composition of natural microbial communities is highly dynamic and time-space dependent, with concomitant short- and long-time phenomena affecting their succession at synoptical and seasonal scales or due to regime shifts (Faust et al., 2015). We suggest that cryopreservation could be one option to control the obvious lack of standardization of the initial composition of complex microbial communities in experimental setups, as it has been shown to be a promising way to preserve both community composition and functioning (Kerckhof et al., 2014).

Cryopreservation is the preservation of biological material at cryogenic ultralow temperatures (−80 or −196°C) for its optimal long-term preservation (Prakash et al., 2013). It is usually carried out with the addition of cryoprotectants, which help to reduce biophysical damage of cells by preventing the formation of intracellular ice crystals, resulting in increased recovery yields upon resuscitation (Fuller, 2004). Among a variety of different cryoprotectants, dimethyl sulfoxide (DMSO) has been described as one of the most successful and commonly used cryoprotectants in microbiology (Hubálek, 2003; Chian, 2010).

The effectivity of cryopreservation has been verified for both axenic and non-axenic cultures during the last decades (Nagai et al., 2005; Heylen et al., 2012a). More recently, it has also been demonstrated for bioreactor communities, which successfully recovered their composition and functionality after cryopreservation (Kerckhof et al., 2014). So far, cryopreservation has been mainly used in applications linked to biotechnology and not yet applied to conserve highly complex and dynamic communities from natural aquatic environments.

Even if cells remain viable after cryopreservation, appropriate thawing and culturing conditions are required to recover the activity of individual community members and effectively resuscitate the preserved communities (Heylen et al., 2012b). However, most prokaryotes occurring in natural habitats have not yet been successfully cultured (Steen et al., 2019), rendering the resuscitation process particularly challenging for cryopreserved natural communities. Shortcomings of prokaryotes culturing can be associated with their narrow and mostly unknown physiological requirements (Rappé et al., 2002; Giovannoni, 2017), such as their adaption to low nutrient levels, the frequent occurrence of temporarily inactive cellular states (i.e., starvation, dormancy), and the complexity of inter-species interactions (Pagnier et al., 2015; Overmann et al., 2017). Widely applied culture media, such as Marine Broth (ZoBell, 1941), are in striking contrast to global ocean nutrient levels and do not represent properly the environmental conditions (Giovannoni and Stingl, 2007).

Culture media based on the original environmental conditions, such as filtered seawater and artificial fresh- or sea-water amended with low concentration of nutrients, have been shown to increase the culturing success (Overmann et al., 2017). It has further been suggested that complex carbon substrates can promote collaborative interactions among species in mixed cultures (Deng and Wang, 2016). However, the extremely high diversity and reactivity of the molecules constituting the natural dissolved organic matter (DOM) (Hansell, 2013) makes its reproduction in laboratory conditions challenging. To tackle this difficulty, representative DOM sources, such as leachates obtained from representative carbon sources (e.g., plant litter or leaves) or artificially created DOM substrates with contrasting chemical reactivities (e.g., monomers vs oligomers) have been applied in earlier studies to assess the effect of several DOM pools on the microbial community composition and activity (Lechtenfeld et al., 2015; D’Andrilli et al., 2019; Morán et al., 2020).

**FIGURE 1:**
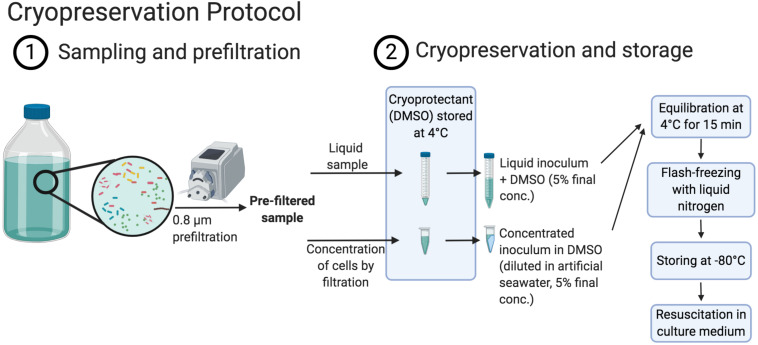
Schema displaying the cryopreservation procedure (created with BioRender.com).

In this study, we propose a method to improve the control of initial microbial communities of experiments through the cryopreservation and resuscitation of natural aquatic microbial assemblages in either sterile filtered seawater or artificial seawater (ASW) with DOM supplements that were prepared from the particulate organic fraction of seawater (Figure 1). The method was tested on communities harvested from a coastal station and a closely located coastal lagoon on the Northwest coast of the Mediterranean Sea, which have contrasting trophic status and environmental variability. More specifically we assessed (i) the impact of inoculum size on the resuscitation and (ii) to which extent diversity and community composition can be maintained according to inoculum characteristics and culture medium. We further evaluated the effect of (iii) handling time during preparation of community aliquots and (iv) storage time of the cryopreserved aliquots on the community composition of the resuscitated communities.

## Materials and Methods

### Sampling Locations

**FIGURE 2:**
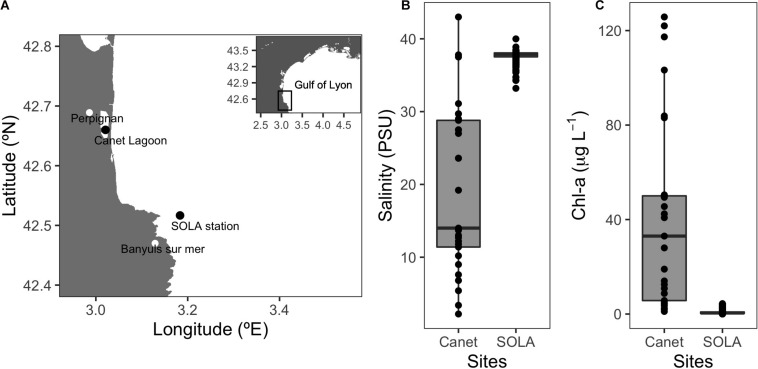
Sampling sites and representative environmental conditions. **(A)** Sampling location of the SOLA coastal station and the Canet Lagoon. **(B)** Salinity (PSU) and **(C)** chlorophyll-a (μg L^–1^) for the Canet lagoon and SOLA station. Salinity and chlorophyll data for SOLA were provided by the SOMLIT program (http://somlit-db.epoc.u-bordeaux1.fr/bdd.php; 03/05/2005-30/10/2018, *n* = 595). Salinity and chlorophyll data for the Canet lagoon were extracted from the IFREMER Surval service (https://wwz.ifremer.fr/surval/, 26/06/2001-07/09/2006, *n* = 87).

Prokaryotic communities were sampled from the SOLA coastal station in the northwest Mediterranean Sea and the Canet Lagoon. Both sites are located on the southern coast of the Gulf of Lyon in the region of Occitanie, South France (Figure 2A and Table 1). These two locations were chosen because of their contrasting environmental conditions: SOLA represents an oligotrophic site with chlorophyll-a (Chl-a) concentrations varying between 0.01 and 4.39 μg L^–1^ with rather stable salinity concentrations (33.20–39.99 PSU) and Canet is a shallow coastal Lagoon characterized by high productivity and large-scale salinity changes (1.09–125.76 μg L^–1^ Chl-a, 2.20–43.00 PSU; Figures 2B,C) over the year.

**TABLE 1:** Overview of sampling locations and dates.

Location	Sampling type	Latitude (°N)	Longitude (°E)	Sampling date
SOLA	Community^a^/DOM^b^	42° 29.0′	03° 08.7′	03/07/2018, 18/02/2020
Canet	Community	42° 40.0′	03° 01.0′	07/08/2018
Warnow	DOM	54°10.2′	12°06.3′	10/08/2017

*^a^Community = samples for cryopreserved communities. ^b^DOM = samples for DOM supplements.*

Dissolved organic matter supplements that served as substrates for the resuscitated communities were obtained from either the SOLA sample site or from the Warnow River Mouth into the Baltic Sea, in Warnemünde, Germany (Table 1), as detailed below. The Warnow River Mouth has a salinity range of 5–18 PSU due to the mixing with water masses from the Baltic Sea (Schernewski et al., 2019). The Warnow estuary is highly productive due to terrestrial loads, with Chl-a values ranging between 18 and 65 μg L^–1^ (Freese et al., 2007).

### Preparation of Culture Media and Dissolved Organic Matter (DOM) Supplements

Either sterile filtered water from the SOLA station, hereafter F-SEA, filtered through 0.22 μm, 47 mm cellulose filter (Millipore, MA, United States) or inorganic ASW basic medium (Eguchi et al., 1996) supplemented with DOM was used as medium. Besides the DOM supplements, no further carbon, nitrogen, phosphorus, or vitamins were added to the ASW medium. Trace metals, Fe, and EDTA were added 100 times less concentrated than in the ASW original recipe (Eguchi et al., 1996), with a final salinity of 35 g L^–1^ and a pH of 8. For the preparation of DOM supplements, water from the SOLA station and the Warnow River were sampled in 25 L polycarbonate carboys previously rinsed with HCl 10% and Milli-Q water. A total of 65 and 13.6 L of the Warnow and SOLA water, respectively, were filtered through 0.22 μm cellulose acetate filters (OE 66, 142 mm, Whatman, Buckinghamshire, United Kingdom) to retain bacteria and all particles in larger size classes. These filters were then stored frozen (−20°C) until further processing.

To prepare the DOM used as supplement, the filters were ground in a stainless-steel mortar and pestle (Bel-Art^TM^, Fisher Scientific, Strasbourg, France) while immersed in liquid nitrogen. Next, the filter pieces were resuspended in a known volume of ultrapure water (60 mL) and autoclaved at 120°C for 20 min. Subsequently, the sterile suspensions containing the filter pieces were filtered through pre-combusted (450°C, 6 h) GF/F 47 mm filters (Whatman, Buckinghamshire, United Kingdom) to remove particles. Finally, the obtained DOM supplements were stored frozen at −20°C until use.

For this study, two kinds of DOM supplements were used: one containing exclusively DOM from SOLA (hereafter S-DOM) and the second containing mixed DOM from SOLA and the Warnow River (hereafter SW-DOM). S-DOM obtained from the equivalent of 0.1 L of sample water was added to 1 L of ASW medium, while DOM supplements from the equivalent of 0.05 L from the sample water obtained from SOLA and 0.17 L from the Warnow River were added to 1 L ASW medium in the SW-DOM medium.

### Chemical Characterization of DOM Supplements

To characterize the DOM supplements, the concentration of dissolved organic carbon (DOC), dissolved organic nitrogen (DON), dissolved organic phosphorous (DOP), and nutrients (NO_3_^–^, NO_2_^–^, and PO_4_^3–^) were determined. Aliquots of the water samples and DOM concentrates were filtered through two pre-combusted (450°C, 6 h) 25 mm GF/F filters (pore size 0.7 μm, Whatman, Buckinghamshire, United Kingdom). Aliquots for DOC measurements (10 mL) were stored in combusted glass ampoules and acidified (85% H_3_PO_4_, final pH = 2). The ampoules were closed and maintained at room temperature until analyzed on a Shimadzu TOC-V following a protocol published elsewhere (Cauwet, 1994). Aliquots for DON and DOP (20 mL) were collected in Teflon bottles and, stored at −20°C. DON and DOP were simultaneously determined using the wet oxidation method (Pujo-Pay and Raimbault, 1994). Nitrate, (NO_3_^–^), nitrite (NO_2_^–^), and phosphate (PO_4_^3–^) were determined by standard colorimetric technique (Bran Luebbe autoanalyzer) (Aminot and Kérouel, 2007).

### Cryopreservation of Natural Prokaryotic Communities

We have adapted the cryopreservation procedure developed by Vekeman and Heylen (2017) for the cryopreservation of single strains or mixed cultures to cryopreserve natural aquatic communities. This previously published protocol had been applied by the authors exclusively to laboratory cell cultures grown in high cell densities (>10^8^ cells/mL, Vekeman and Heylen, 2017) while cell densities in marine bacterial communities range typically from 10^5^ to 10^6^ cells/mL (Kirchman, 2008). For this reason, but also to minimize the carryover of substrates to new culture media, a step to concentrate the cells present in aquatic samples before cryopreservation was added.

Previous to the cryopreservation protocol and in order to remove potentially present bacterivorous protists, which may also be cryopreserved in DMSO (Supplementary Figure S1; Liu et al., 2009), the sample water was pre-filtered using 0.8 μm pore size filters (47 mm mixed cellulose esters, Millipore, MA, United States). We have performed this prefiltration step as it is a common practice in experimental work with natural aquatic microbial assemblies, to avoid protist grazing during incubations (e.g., Giorgio et al., 1996; Beardsley et al., 2003; Szekely et al., 2013; Kujawinski et al., 2016). However, studies with a focus only on the first incubation days before protists start to proliferate (approximately 4 days after prokaryotes start to proliferate, Supplementary Figure S1), or that are particularly interested in incubations containing protists, may skip the prefiltration step.

Between 0.1 and 1.5 L of pre-filtered water was subsequently filtered through 0.22 μm filter (hydrophilic PVDF, 25 mm filter, Millipore, MA, United States) using a vacuum pump (∼600 mg Hg) in order to concentrate prokaryotic cells. Drying of the filters was carefully avoided during the filtration, except at its end, when the pressure was set to a minimum, to prevent cell damage, and the final water volume covering the filter was slowly filtered. Immediately after removal of the sample water, the pump was turned off and 0.5 mL of inorganic ASW medium was added to the top of the filter, which was then used to resuspend the cells from the filter by pipetting up and down. This volume was subsequently transferred into previously prepared 2 mL tubes with 0.5 mL cryoprotectant containing 10% (v/v) sterile-filtered DMSO (ACS reagent, 99.9%, Sigma–Aldrich, St. Louis, MO, United States) in inorganic ASW, equilibrated at 4°C (DMSO final concentration: 5%). Next, the filters were completely immersed into the medium with cryoprotectant and resuspend cells and the tubes were kept at 4°C for 15 min for equilibration (Vekeman and Heylen, 2017). The tubes were then flash-frozen in liquid nitrogen and transferred to −80°C for long-term storage.

We additionally prepared 10 mL aliquots of 0.8 μm pre-filtered water samples without prior cell concentration via filtration. Sterile-filtered DMSO was added as cryoprotectant (5% final concentration). Then, the aliquots were kept for 15 min at 4°C before flash-freezing in liquid nitrogen and transferred to −80°C for long-term storage (Figure 1; for a step-by-step protocol, see Supplementary Text S1).

The abundance of prokaryotic cells in the 0.8 μm pre-filtered inoculum water of SOLA and Canet was estimated via flow cytometry as detailed elsewhere (Marie et al., 2000). In short, aliquots (1350 μL) were fixed with glutaraldehyde (0.1% final concentration) and stored at −80°C until analysis. Samples were analyzed using either the cytometer FACSCanto II (BD Biosciences, Franklin Lakes, NJ, United States) or the Cytoflex Flow Cytometer (Beckman Coulter, Brea, CA, United States) (Table 2).

**TABLE 2:** Overview of the performed experiments.

ID experiment	Description	Inoculum source	Inoculum size (10^6^ cells)	Temperature (°C)	Growth medium	Inc. time (days)	Variables analyzed
1a	Effect of the inoculum size (filters)	SOLA	66, 132, 330, 495, 990	15	S-DOM	6	Microbial abundance^b^
1b	Effect of the inoculum size (water aliquot)	SOLA	1.32, 3.30, 6.60, 9.90	15	S-DOM	6	Microbial abundance^b^
2	Similarity between resuscitated and original communities (filters)	SOLA, Canet	SOLA: 412, Canet: 804	18	F-SEA, S-DOM, SW-DOM	5	Microbial abundance^a^, community composition (16S rRNA sequencing)
3	Handling time (filters)	SOLA	412	18	S-DOM	6	Microbial abundance^a^,
							Community composition
							(ARISA fingerprinting)
4	Storage time (filters)	SOLA	412	18	S-DOM	5	Microbial abundance^b^,
							Community composition
							(ARISA fingerprinting)

*^a^Flow cytometer FACSCanto II (BD Biosciences). ^b^Flow cytometer Cytoflex (Beckman Coulter).*

Additionally to the preparation of filters with cryopreserved communities, 1.5 L of the 0.8 μm (47 mm mixed cellulose esters, Millipore, MA, United States) pre-filtered water from both locations was filtered onto 0.22 μm filters (47 mm cellulose filters, Millipore, MA, United States) for later DNA extraction and 16s rRNA gene sequencing to characterize the initial communities.

### Resuscitation of Natural Prokaryotic Communities and Experimental Designs

To resuscitate the cryopreserved communities, the cryopreserved samples were thawed at room temperature (∼30 min) and then added (together with the filter where applicable) to the culture medium. The cells were incubated for 5–6 days close to the *in situ* temperature (15–18°C, Table 2) in glass bottles under agitation with magnetic stirrers. In total, four experiments were performed to test and evaluate the cryopreservation and resuscitation of the natural aquatic prokaryote communities (Table 2). In all resuscitation experiments, cell numbers were monitored daily via flow cytometry as detailed above.

In the first experiment, we assessed the effect of initial cell numbers on cell growth after resuscitation, including both cryopreserved filter-concentrated inocula (experiment 1a), and cryopreserved water aliquots from 0.8 μm pre-filtered SOLA seawater (experiment 1b). For experiment 1a, filters containing concentrated cryopreserved communities were prepared from 100, 200, 500, 750, and 1500 mL 0.8 μm pre-filtered SOLA seawater. These inocula contained, according to the results from flow cytometry analyses, 66, 132, 330, 495, and 990 million cells in total. Triplicate cryopreserved communities of each volume were resuscitated in specific volumes of S-DOM medium (60, 120, 300, 450, and 900 mL, respectively) in order to ensure constant initial cell abundance in the incubations (1.1 × 10^6^ cells/mL). For experiment 1b, cryopreserved water aliquots (2, 5, 10, and 15 mL) from the 0.8 μm prefiltered SOLA seawater were diluted 10-fold and resuscitated in triplicates in S-DOM medium (initial abundance of 0.07 × 10^6^ cells/mL). The starting inocula in this experiment contained 1.32, 3.30, 6.60, and 9.90 million cells, respectively. The resuscitated cells in both experiments were incubated for 6 days (Table 2). Since larger inocula resulted in improved similarity among resuscitation replicates, all downstream experiments were performed using filter-concentrated inocula containing ≥412 × 10^6^ cells.

Next, experiment 2 was performed with the aim to evaluate the similarity of resuscitated communities to the initial starting communities in dependence of the growth medium and inoculum source. Cryopreserved filter-concentrated inocula from SOLA (4.12 × 10^8^ cells) and Canet (8.04 × 10^8^ cells) were incubated in 900 mL medium in 1 L Duran Schott bottles. Triplicate SOLA inocula were incubated in F-SEA, ASW supplemented with S-DOM extract, and ASW supplemented with SW-DOM extract. Triplicate Canet inocula were incubated only in ASW supplemented with S-DOM or SW-DOM. The water of the F-SEA treatment was prepared at the same day as the cryopreserved SOLA community and inoculated on the evening of that same day after the communities had been cryopreserved for a few hours. This was done to avoid or minimize chemical changes in the F-SEA medium that could occur during prolonged storage time. The incubation of SOLA communities in F-SEA was performed to test the resuscitation procedure in an environment as close as possible to the natural environment of the initial SOLA community. Incubations in the S-DOM and SW-DOM media differed in their C:N:P ratio (Table 3) and represent media with different trophic status. Not surprisingly, the C:N:P ratios of S-DOM and SW-DOM were similar to the C:N:P ratios of cell contents reported in previous reports (45:9:1; Goldman et al., 1987), while F-SEA was more depleted in both nitrogen and phosphorous. The communities reached stationary phase on the fifth day, when they were collected onto 0.22 μm filters (Millipore, MA, United States) for later DNA extraction and 16s rRNA gene amplicon sequencing.

**TABLE 3:** Characterization of DOM supplements used as substrates in the resuscitation experiments.

	F-SEA (μmol L^–1^)	S-DOM (μmol L^–1^)	SW-DOM (μmol L^–1^)
DOC	78.20^a^	0.708	1.952
DON	6.52^b^	0.046	0.524
DOP	0.07^b^	0.011	0.071
C:N:P	1050:84:1	60:4:1	25:7:1

*^a^Concentration corresponds to July 2013. Data provided by Sánchez-Pérez et al. (2020). ^b^Estimation based on the Redfield ratio described by Pujo-Pay et al. (2011) for the western surface Mediterranean waters.*

The impact of the handling time to prepare cryopreserved community aliquots and the impact of the storage time of these aliquots on the resuscitated communities were tested in experiments 3 and 4, respectively.

In experiment 3, a set of triplicate filter-concentrated inocula from SOLA was cryopreserved immediately after the pre-filtration step and a further set of that same community was cryopreserved 7.5 h later. Cryopreserved communities from both sets (4.12 × 10^8^ cells) were resuscitated in 900 mL of S-DOM medium. In experiment 4, three sets of cryopreserved filter-concentrated communities from SOLA (4.12 × 10^8^ cells) were resuscitated after cryopreservation for 1.5, 6, and 12 months. The resuscitation was performed in 900 mL S-DOM medium in 1 L Duran Schott bottles. In both experiments, the communities were incubated for 5–6 days (Table 2), before they were collected onto 0.22 μm pore size filters (Millipore, MA, United States) for later DNA extraction and 16s rRNA gene fingerprinting via Automated Ribosomal Intergenic Spacer Analysis (ARISA) (Cardinale et al., 2004).

### DNA Extraction

Filters for DNA extractions were stored at −30°C. DNA extraction was performed after cutting the filters in small pieces and using a modified protocol of the QIAmp DNA Mini Kit (QIAGEN, Hilden, Germany) with an initial bead-beating step in ATL buffer using a FastPrep-24^TM^ 5G (MP Biomedicals, Irvine, CA, United States). Concentration and quality of the eluted DNA were tested by electrophoresis (1% agarose gel) and Quantus fluorometer using the PicoGreen assay (Promega, Madison, WI, United States).

### 16s rRNA Gene Amplicon Sequencing and Bioinformatic Evaluation

DNA samples from the final incubation day of experiment 2 and from the initial 0.8 μm pre-filtered SOLA and Canet communities were sent for 16s rRNA gene amplicon sequencing (300 base pairs paired-end read, Illumina Miseq V3) using the primers pair 515yf-926r (Parada et al., 2016). For the SOLA and Canet initial communities, only one DNA extract from each was available and we performed triplicate library preparation in order to obtain replicates that reflect the technical variance introduced during library preparation and sequencing. Demultiplexing of libraries via the Illumina bcl2fastq 2.17.1.14 software, removal of reads with length <100 bases, as well as primer clipping (up to three mismatches per primer), were performed by the sequencing company (LGC Genomics GmbH). Further processing of the sequence reads was done in R using the DADA2 pipeline (Callahan et al., 2016) applying settings given in the instructions^1^, with some modifications (R-code is available in https://github.com/angelrainf/gesifus.cryo.dada2). A total of 700 amplicon sequence variants (ASVs) were identified across all sequenced samples and the obtained ASVs were taxonomically assigned using the Genome Taxonomy Database (GTDB) (Parks et al., 2018). Raw sequence data are available at European Nucleotide Archive under the accession number PRJEB38947.

### ARISA Fingerprinting

Differences in the community composition of incubations in experiments 3 and 4 were estimated using ARISA analysis. Extracted DNA from the communities was amplified with KAPA2G Fast Ready Mix (Sigma–Aldrich, St. Louis, MO, United States) in 20 μL reactions using 10 μM of the primers 1406F and 23SRY (Frossard et al., 2012) and 1 μL of DNA template. Reactions mixtures were initially held at 94°C for 3 min, followed by 35 amplification cycles of 94°C for 15 s, 55°C for 15 s, and 72°C for 30 s, with a final extension of 72°C for 1 min.

PCR products were subsequently purified through Sephadex G-50 fine (Sigma–Aldrich, St. Louis, MO, United States) and quantified with a Quantus fluorometer with the Picogreen assay (Promega, Madison, WI, United States). Then, 2.5 μL of purified PCR products was mixed with 0.2 μL of MapMarker1000 (Bioventures, Murfreesboro, TN, United States) and 15 μL of formamide (Sigma–Aldrich, St. Louis, MO, United States) and, denatured at 95°C for 3 min. Samples were then sequenced in a Sequencer 3130XL (Applied Biosystems^®^, Foster City, CA, United States).

Terminal restriction fragments (T-RFs) were identified using the GeneMapper software (Applied Biosystems^®^, Foster City, CA, United States) and the areas of the identified T-RF peaks were imported into the T-Rex (T-RFLP analysis EXpedited) online software for noise filtering, alignment, and binning by default parameters (Culman et al., 2009). ARISA data were normalized by dividing the area of the individual peak by the total peak area of the sample.

### Statistical Analysis and Data Acquisition

All statistical analyses were performed using the R platform (R Core Team, 2018) employing the packages vegan (Oksanen et al., 2019), lme4 (Bates et al., 2015), and lmerTest (Kuznetsova et al., 2017). Sample variation descriptors given after mean values correspond to the standard deviation if not otherwise specified.

For describing the growth curves during incubations, carrying capacity was estimated by fitting a logistic growth equation (García et al., 2018). To test the impact of inoculum size on the variability of cell growth after resuscitation (experiments 1a,b), we estimated the variance and the coefficient of variation (CV) of the cell abundances of resuscitated inocula after 4, 5, and 6 days of incubation. We fitted a mixed linear model for both the variance and the CV, using the incubation day as random factors and the inoculum sizes as fixed factors. The CV of cell abundance was estimated for experiments 2, 3, and 4 from the time point with maximum abundance. We further compared the variability of cell growth among simultaneously performed triplicate incubations in our study with triplicate incubations of diluted, non-cryopreserved natural aquatic microbial communities published elsewhere (Morán et al., 2020). For this purpose, CV values from the latter incubations were estimated from digitalized figures in Morán et al. (2020) by the Engauge Digitizer Program (Mitchell et al., 2019).

In experiment 2, we compared initial communities of SOLA and Canet with resuscitated communities after several days of incubation. However, while triplicate values in the initial communities represent technical replicates, triplicates after resuscitation represent biological replicates with presumably larger variance. Differences in variance among compared datasets violate assumptions for two-sample *t*-tests. We accordingly used a one-sample *t*-test (two-tailed) to statistically compare the Shannon diversity between initial and resuscitated communities. For this purpose, we used the mean Shannon diversity of the technical replicates from the initial community as a reference value and we subtracted Shannon diversities from the triplicate incubations from this mean. The one-sample *t*-test was then applied to test the null hypothesis that the difference in diversity between the initial sample used as inoculum and the incubations did not differ from zero.

To assess and subsequently visualize the similarity of resuscitated communities to the initial starting communities in dependence of the growth medium and inoculum (experiment 2), pairwise Bray–Curtis distance indices between all communities were calculated. This was done based on the relative abundance of ASVs in each community obtained after rarefication to the minimum number of processed reads obtained for the individual libraries (Canet initial, 13,200 reads, Supplementary Table S1).

We further evaluated the number and proportion of initially abundant ASVs (>0.5% relative abundance in the initial pre-filtered samples of SOLA and Canet) that exhibited cell growth and that were thus viable during the incubations. In this analysis, we compared absolute abundance estimates of individual ASVs in the initial communities with estimates obtained from the resuscitated communities. Absolute abundances of individual ASVs were calculated by multiplying the relative abundances of each ASV by the total cell counts of the communities from which they were identified. Because of the biases during DNA extraction and/or amplification, this is not a proper estimation of the absolute cell numbers of individual ASVs within a community (Andersson et al., 2010). However, the dynamics of absolute cell numbers, such as the fold change, and thus, the cell growth of specific ASVs between two communities can be approximated because the biases among compared samples are similar (Props et al., 2017; Ward et al., 2017; Shen et al., 2018a).

Log2-fold-change (L2FC) of the absolute abundances of each ASV exceeding 0.5% relative abundance in the initial sample between the incubation start (day 0) and the final incubation day was used to estimate their growth in each incubation. Positive L2FC values indicate that the tested ASV exhibited more cells in the incubation medium after 5 days than in the starting community, implying that cells affiliating with this ASV were actively replicating.

To statistically evaluate the growth of individual ASVs originating from the SOLA and Canet inocula after the cryopreservation and resuscitation in the different media, we applied similarly as above a one-sample *t*-test (two-tailed) to the replicate L2FC values of each AVS per treatment (SOLA: F-SEA, S-DOM, SW-DOM; Canet: S-DOM, SW-DOM). In the F-SEA incubations, we omitted one of the three replicates from the *t*-test, where the community was different from the other two replicates due to the overgrow of members of the Enterobacterales order. ASVs with (significantly) positive L2FC in at least one medium were considered as viable. *P*-values were adjusted with the Benjamini–Hochberg correction to account for false discovery rates during multiple comparisons (Benjamini and Hochberg, 1995).

To visualize the effect of handling and storage time, Hierarchical Clustering (Ward.D2) was performed on the normalized ARISA community fingerprint data based on Bray–Curtis distances from the incubations obtained in experiments 3 and 4. Analyses of similarities (ANOSIM, permutations = 999) were performed to evaluate the effect of the handling and storage time. In the case of the storage time analysis, an additional *post hoc* pairwise ANOSIM was performed between each of the three tested storage times followed by a Bonferroni correction for multiple comparisons. For the samples of experiment 4 (storage time), we furthermore applied a Mantel test between the Bray–Curtis distance matrix and the Euclidean distance matrix of storage time to evaluate the effect of storage time on the dissimilarity among samples.

## Results

### Effect of the Inoculum Size on the Resuscitation Procedure (Experiment 1a/b)

**FIGURE 3:**
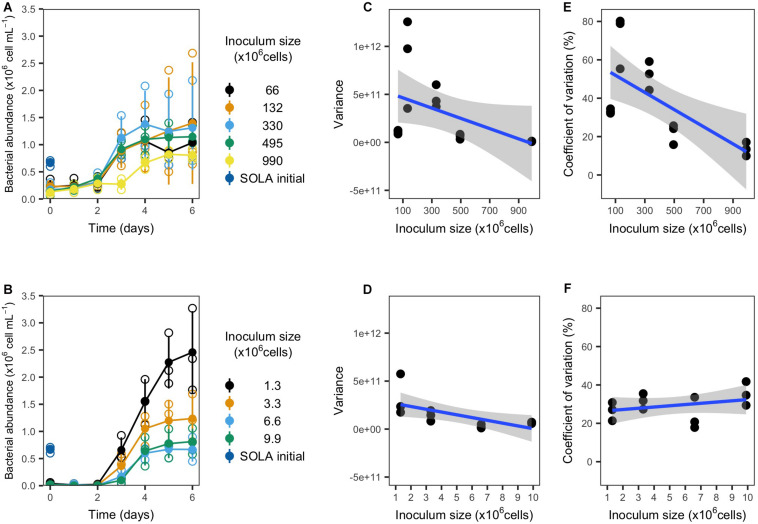
Growth and variability of resuscitated communities from experiment 1a (filter concentrated inocula; upper panels) and 1b (water inocula; lower panels). **(A,B)** Growth curves. Empty circles represent the individual replicates, while filled circles represent the replicates mean (*n* = 3). **(C,D)** Variance and **(E,F)** coefficient of variation for bacterial cells detected after 4, 5, and 6 incubation days in dependence on the inoculum size in triplicate incubations.

The resuscitation and subsequent incubation of cryopreserved inocula in S-DOM media succeeded independently from the inoculum type (concentrated by filtration or sample water) and inoculum size (Figures 3A,B). Both inoculum types displayed longer lag-phases (3 days) for the largest inocula, while the lag phase did not exceed 2 days in all remaining treatments. Furthermore, the carrying capacity of all incubations surpassed the cell numbers detected in the initial 0.8 μm pre-filtered SOLA water (0.66 ± 0.05 × 10^6^ cells/mL), and the difference was generally greater in incubations with smaller inoculum, except for the smallest inoculum in experiment 1a (Table 4).

**TABLE 4:** Carrying capacities and statistical parameters for experiment 1.

Experiment	Inoculum size (10^6^ cells)	Carrying capacity (×10^6^ cells/mL)	Mixed linear model		
			Variable	Slope^a^	*P*
1a) Filter-concentrated inocula	66	0.99 ± 0.31	Variance	−5.35 × 10^8^	0.081
	132	1.51 ± 1.27		(±2.77 × 10^8^)	
	330	1.34 ± 0.68	CV	−0.04 (±0.01)	0.025
	495	1.17 ± 0.25			
	990	0.99 ± 0.14			
1b) Water inocula	1.32	2.49 ± 0.68	Variance	−2.89 × 10^10^	0.168
	3.3	1.22 ± 0.42		(±1.42 × 10^10^)	
	6.6	0.67 ± 0.17	CV	0.65 (±0.63)	0.355
	9.9	0.80 ± 0.25			

*^a^Numbers in brackets indicate the standard error.*

We observed for both inoculum types that the variance among replicates tended to decrease with increasing inoculum size (Figures 3C,D). Moreover, we observed a significant decrease in the CV for cryopreserved filter inocula. In contrast, water inocula incubations displayed a trend for the CV to increase with larger inocula (Figures 3E,F and Table 4).

### Community Similarity of Initial and Resuscitated Communities in Dependence of Media and Inoculum Source (Experiment 2)

**FIGURE 4:**
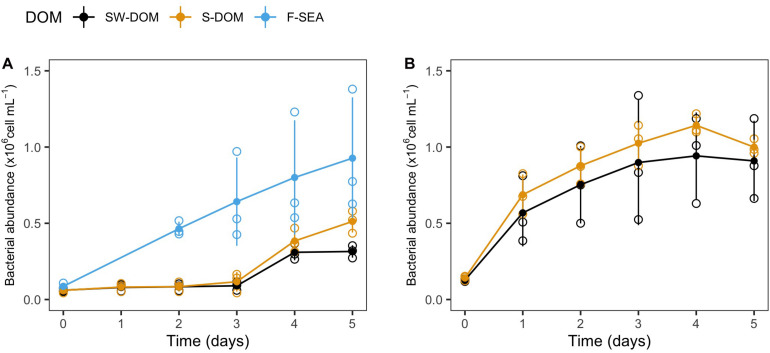
Growth of resuscitated communities incubated in different media from experiment 2. Growth curves of resuscitated communities originating from **(A)** SOLA and **(B)** the Canet lagoon. Error bars represent standard deviation among replicates (*n* = 3). Empty circles illustrate the individual replicates, while filled circles represent the mean of triplicate incubations.

Cryopreserved communities from the SOLA field station and Canet that were resuscitated in experiment 2 exhibited growth in all tested media (F-SEA, S-DOM, and SW-DOM, Figure 4). The carrying capacity of SOLA resuscitated communities in F-SEA was highly variable (0.93 ± 0.42 × 10^6^ cells/mL) compared to the S-DOM (0.80 ± 0.26 × 10^6^ cells/mL) and SW-DOM (0.43 ± 0.13 × 10^6^ cells/mL), a difference caused by one of the F-SEA triplicate incubations reaching much higher abundances than the other two replicates (Figure 4A). The incubations of Canet cryopreserved communities exhibited carrying capacities of 1.05 ± 0.07 × 10^6^ and 0.92 ± 0.29 × 10^6^ cells/mL in S-DOM and SW-DOM medium, respectively, with larger differences among the replicates in the SW-DOM medium (Figure 4B). Lag-phases lasting for 3 days were observed in the resuscitated SOLA communities incubated in the S-DOM or SW-DOM media, while lag-phases were detected neither for the resuscitated SOLA communities growing in the F-SEA medium nor for the resuscitated Canet communities growing in both tested media (Figure 4).

**FIGURE 5:**
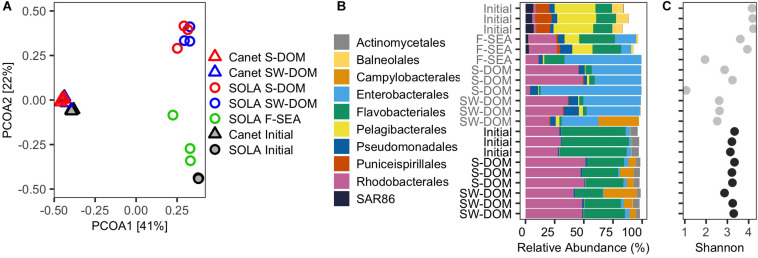
Initial and resuscitated microbial assemblies from experiment 2. **(A)** PCoA biplot displaying the community similarities (based on ASVs) among the initial and resuscitated communities in different media. **(B)** Relative contribution of the 10 most abundant orders in the initial communities as well as in the resuscitated communities after 5 days incubation in different media. **(C)** Shannon diversity indices. **(B)** and **(C)** share the same *y*-axis labels.

The ASV composition of the F-SEA treatment was moderately similar to the initial SOLA community in two replicates (average Bray–Curtis dissimilarity: 0.62 ± 0.08, Figure 5A), while the replicate characterized by high cell numbers differed at a higher degree (Bray–Curtis dissimilarity: 0.93, Figure 5A). Also, S-DOM and SW-DOM treatments resulted in high dissimilarity relative to the initial community (average Bray–Curtis dissimilarity: 0.96 ± 0.01 and 0.92 ± 0.02, respectively, Figure 5A). In contrast, resuscitated Canet communities exhibited comparatively high similarity to the initial Canet community regardless of the growth medium used, with an average Bray–Curtis dissimilarity of 0.50 ± 0.04 and 0.52 ± 0.01 for S-DOM and SW-DOM, respectively (Figure 5A), even though the DOM supplements did not originate from the Canet Lagoon (S-DOM, SW-DOM).

There were considerable similarities between the community composition of two replicates of the F-SEA communities, at the order level, and the initial SOLA community (Figures 5A,B). However, members of the Pelagibacterales, which represented approximately 34% of all ASVs in the initial SOLA community, were particularly underrepresented in these two F-SEA incubations (∼15%). Likewise, members of the orders Balneolales, Puniceispirillales, and SAR86 clade that represented between 7 and 13% decreased to 1.6–2.7%. Conversely, members of the orders Flavobacteriales, Rhodobacterales, and Pseudomonadales were overrepresented in these two F-SEA incubations (Figure 5B). Members of the Enterobacterales, mostly affiliated with the genus Alteromonas (Supplementary Table S2), displayed low abundance in the initial SOLA community (∼0.03%) but increased substantially and even dominated in one of the incubations (Figure 5B).

The trends in compositional change at the order level described here for the two F-SEA incubations with high similarity to the initial SOLA samples were also observed, at a more pronounced level, for the SOLA community incubated in S-DOM or SW-DOM media, as well as for one of the F-SEA incubations. In one of the SW-DOM incubations, we additionally detected members of the order Campylobacterales that were not detected in any of the other SOLA communities. The detection of Campylobacterales in a single SOLA SW-DOM incubation may have resulted from contamination from the Canet communities, where Campylobacterales were abundant.

Resuscitated Canet communities remarkably resembled the initial Canet community at the order level, regardless of the medium used (Figure 5C). Members of Actinomycetales and Flavobacteriales orders, which represented around 6–56% of all the ASVs in the initial community, decreased to ∼3 and 30% in the resuscitated S-DOM and SW-DOM incubations, respectively. In contrast, Rhodobacterales increased their abundances in all the resuscitated treatments, from ∼29% in the initial Canet community to ∼48%. Strikingly, Campylobacterales members that affiliated exclusively with the genus Arcobacter (Supplementary Table S2) were present in low abundances in the initial Canet community (∼0.7%) and increased to up to 28% abundance in the resuscitated communities. Members of the Enterobacterales order were similarly represented in both the initial and resuscitated communities (∼3%).

SOLA communities resuscitated in the F-SEA medium exhibited two incubations with Shannon entropy values that were very similar to those estimated for the initial SOLA community (one-sample *t*-test, *P* = 0.238, Figure 5C). For resuscitation of SOLA inocula in S-DOM, no significant differences relative to the initial SOLA community Shannon diversity were detected (one-sample *t*-test, *P* = 0.113). On the other hand, the resuscitation of SOLA inocula in SW-DOM resulted in a significant decrease in Shannon diversity compared to the initial SOLA community (one-sample *t*-test, *P* < 0.001, Figure 5C). Similar levels of Shannon diversity were found in all resuscitated Canet inocula in comparison to the initial Canet community (one-sample *t*-test, *P* = 0.489, and *P* = 0.591 for S-DOM and SW-DOM, respectively, Figure 5C).

**FIGURE 6:**
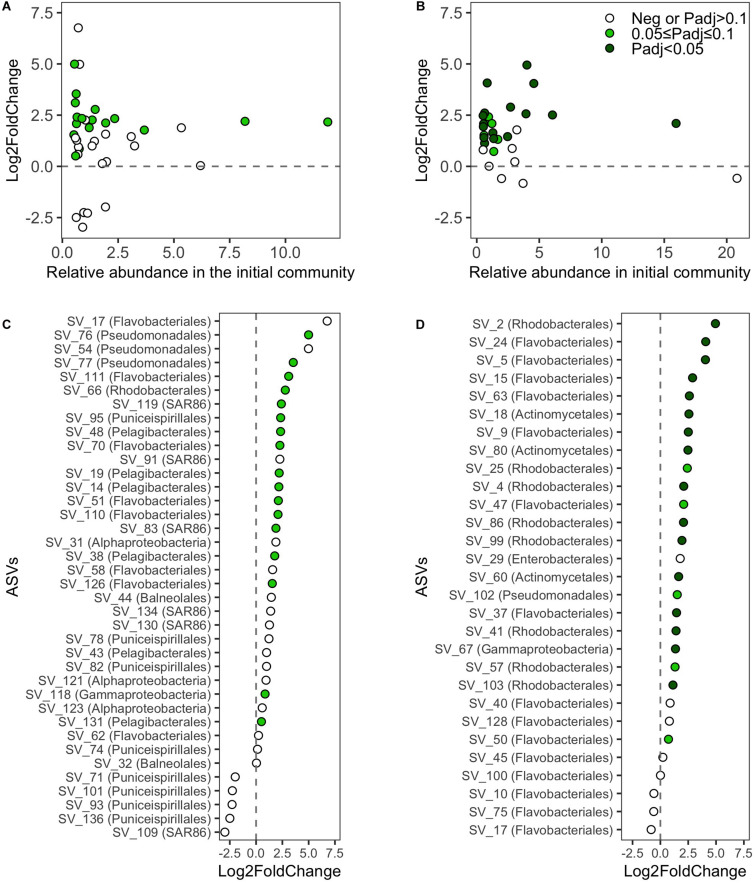
Log2-fold-changes (L2FC) of absolute abundances of ASVs (>0.5% relative abundance in the initial inoculum) between the initial inoculum and the final incubation day. L2FC values of ASVs against their relative abundances in the initial sample from **(A)** SOLA and **(B)** Canet, respectively. Ranked L2FC values of ASVs and the assigned taxonomy at the order level if available or otherwise at the class level for **(C)** SOLA and **(D)** Canet, respectively. For each ASV, only the L2FC in one medium is displayed: we selected for each ASV among the media with positive L2FC the one exhibiting the highest statistical support for growth (lowest *P*-value). For ASVs that exhibited in all media exclusively negative L2FC values, we displayed the one with the lowest *p*-value. Negative L2FC values or positive L2FC values with *P*adj > 0.1 are displayed in empty circles. Positive L2FC values with 0.05 ≤ *P*adj < 0.1 are displayed in light green and positive L2FC values with *P* < 0.05 are displayed in dark green.

Growth analyses of the initially abundant SOLA ASVs in the resuscitated communities revealed that at least 45% (one sample *t*-test, adjusted *P*-value cutoff: 0.1) were viable (Figure 6A). Among ASVs that were initially abundant in the Canet community, at least 72% (one-sample *t*-test, adjusted *P*-value cutoff 0.1) were viable (Figure 6B). ASVs in the SOLA communities for which no active growth could be verified included representatives of the class Puniceispirillales as well as members of the orders Flavobacteriales, Balneolales, the SAR86 clade, and members of the class Alphaproteobacteria (Figure 6C). In the Canet resuscitations, ASVs affiliating with the Flavobacteriales order did not exhibit detectable growth (Figure 6D).

### Effect of Handling Time on the Resuscitation of Cryopreserved Prokaryotic Communities (Experiment 3)

**FIGURE 7:**
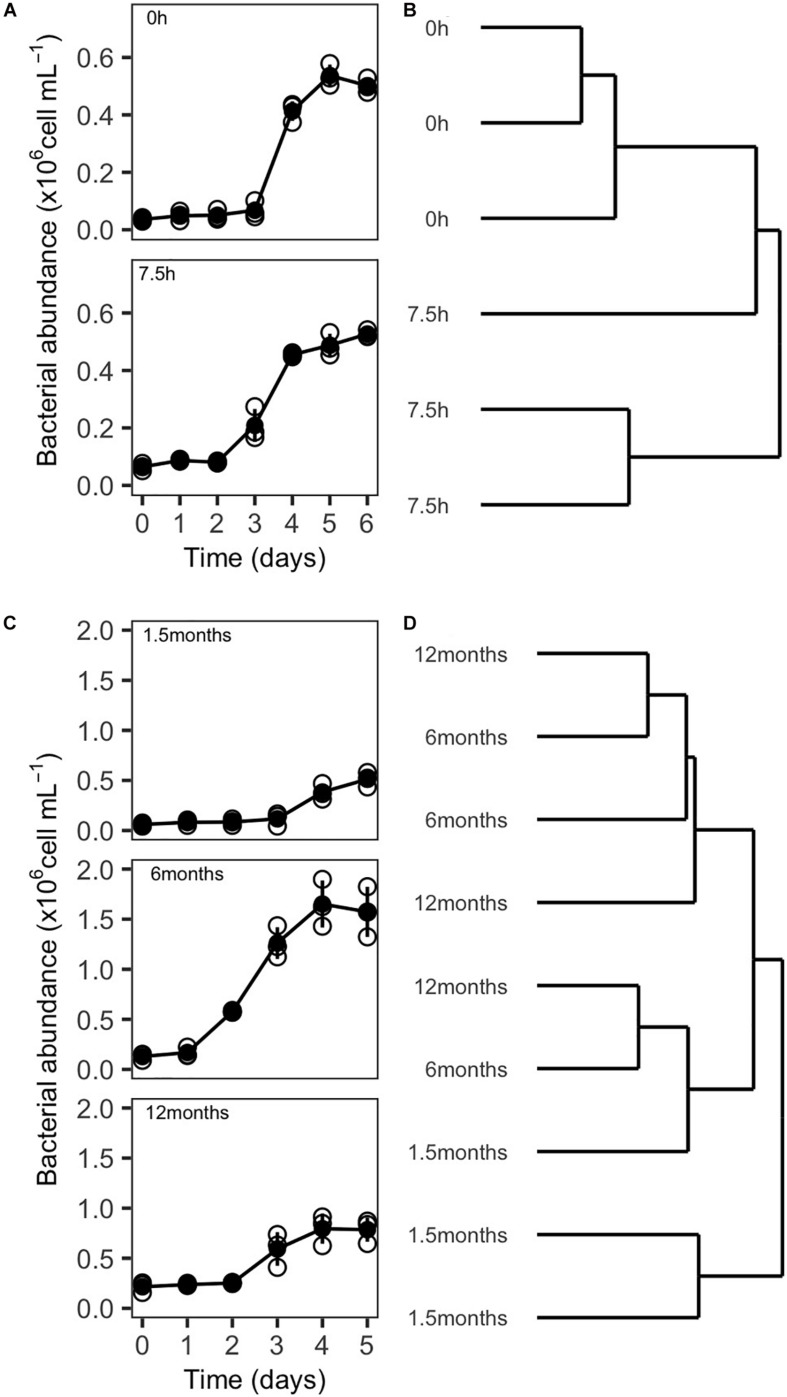
Growth of resuscitated communities and ARISA-based dendrogram displaying community composition similarity from experiment 3 and 4. **(A)** Growth curves and **(B)** dendrogram illustrating the influence of handling time (0 and 7.5 h) during the preparation of inocula for cryopreservation on community composition 5 days after resuscitation. **(C)** Growth curves, and **(D)** dendrogram illustrating the influence of storage time of cryopreserved inocula at –80°C (1.5, 6, and 12 months) on community composition 5 days after resuscitation. Empty circles in **(A)** and **(C)** indicate the individual replicates, while filled circles represent the mean value. Vertical lines represent the standard deviation.

Resuscitated SOLA communities that were processed either directly after the pre-filtration step or after a delay of several hours exhibited similar growth curves with a lag phase of 2–3 days. They also reached similar carrying capacities, varying between 0.52 ± 0.01 × 10^6^ and 0.54 ± 0.02 × 10^6^ cells/mL for 0 and 7.5 h treatment, respectively (Figure 7A).

Differences in community composition between the resuscitated communities were assessed by ARISA fingerprinting, and the hierarchical cluster analysis revealed that communities that had been processed directly after the pre-filtration step clustered closely together (Bray–Curtis distances 0.362 ± 0.074). However, the composition of communities originating from cryopreserved inocula processed with delay was less similar (Bray–Curtis distances 0.741 ± 0.246). Two replicates clustered closely together while the community composition of the third replicate seemed to be more similar to communities from the other incubations (Figure 7B). Further, the ANOSIM did not indicate significant differences in the community composition in dependence of the handling time at an alpha error level of 5% (Table 5).

**TABLE 5:** Statistical parameters from ANOSIM and Mantel tests for experiment 3 and 4.

Experiment	Comparison	Statistical results	*P*
Experiment 3 (handling time)	ANOSIM (all samples)	*R* = 0.333	0.100
Experiment 4 (storage time)	ANOSIM (all samples)	*R* = 0.276	0.069
	ANOSIM (1.5 vs 6 months)	*R* = 0.482	0.300^a^
	ANOSIM (1.5 vs 12 months)	*R* = 0.370	0.300^a^
	ANOSIM (6 vs 12 months)	*R* = 0.000	1.000^a^
	Mantel test	*R* = 0.203	0.117

*^a^P-values were adjusted for multiple pairwise testing via the Bonferroni correction.*

### Effect of Storage Time on the Resuscitation of Cryopreserved Prokaryotic Communities (Experiment 4)

Growth curves of cryopreserved communities that were resuscitated after a storage time of 1.5 and 12 months were similar, with lag phases of 3–4 days and carrying capacities between 0.65 ± 0.14 × 10^6^ and 0.91 ± 0.23 × 10^6^ cells/mL. However, because of a slightly longer lag phase of communities originating from inocula stored for 1.5 months, the communities were, in this case, possibly sampled just before reaching the plateau phase. Incubations from aliquots with a storage time of 6 or 12 months were in contrast sampled shortly after reaching the maximal cell densities (Figure 7C). Different than the other treatments, cryopreserved communities that were resuscitated after 6 months revealed a lag phase of only 1 day and a higher carrying capacity (1.66 ± 0.21 × 10^6^ cells/mL).

However, these differences in the growth curves of the storage time of 6 months vs storage times of 1.5 or 12 months were less evident in the community composition. Here, three clusters in the hierarchical cluster analysis could be observed: one cluster contained two replicates from inocula stored for 1.5 months. A second cluster contained one replicate of each storage time. The third cluster contained two replicates from inocula stored for 6 and 12 months, respectively (Figure 7D). ANOSIM analyses did not reveal significant differences in community composition among samples with different storage time (Table 5). However, the reported significance level (*p* = 0.07, Table 5) was close to the *p*-value cutoff of *p* < 0.05 and could point to an effect of the storage time on community composition. *Post hoc* pairwise comparisons indicated that possible differences in the community composition occurred primarily between the samples with a storage time of 1.5 months vs the samples with 6 and 12 months of storage. Pairwise comparison between samples with 6 and 12 months storage time did not indicate any influence of storage time on community composition in this case. Furthermore, a mantel test did not support a significant increase in community dissimilarity with increasing storage time of the cryopreserved inocula (Table 5).

## Discussion

The cryopreservation of microorganisms has been considered common practice for the long-term storage of isolated strains and, more recently, of relatively simple and usually artificial prokaryotic assemblages. However, cryopreservation techniques for the preservation of complex natural microbial communities have, to our knowledge, not yet been applied.

Experimental setups based on freshly sampled natural microbial communities lack reproducibility in follow-up experiments because the starting community cannot be conserved. We suggested that the cryopreservation of natural prokaryotic communities could circumvent the caveats presented by the lack of standardized initial communities. To the best of our knowledge, this is the first study that tested and evaluated protocols for cryopreservation and resuscitation of highly complex natural prokaryotic assemblages from aquatic habitats. Here, we were able to cryopreserve and resuscitate two distinct and naturally occurring communities preserving most of their diversity.

We interpreted the general trend of lower cell number variances with increasing inoculum size (Figures 3B,D) and a significant decrease in the CV in experiment 1a (filter-concentrated inocula; Figure 3C) as indication that the variability among replicates of the cryopreservation-resuscitation process decreased with increasing inoculum size. The opposite trend for the CV in experiment 1b (not concentrated inocula, Figure 3F) resulted apparently from the increase of both the cell numbers and their variance in the incubations with smaller inocula sizes. These parameters are effectively counteracting each other, since the CV is the standard deviation normalized by the mean of the measured variable.

Increased cell growth and biomass production for smaller inocula, and corresponding lowered biodiversity, contradicts general predictions and observations in BEF research (Yachi and Loreau, 1999; Duffy et al., 2017). Possibly, in the specific case of experiment 1b, the reduction of biodiversity below diversity levels in experiment 1a caused the removal of antagonistic species interactions: fast growers that were suddenly under less competitive pressure may have caused the observed biomass increase in incubations with small inocula and, accordingly, low diversity. The reduction of species diversity in small inocula may further be a reasonable explanation for higher variability of cell growth during the incubations.

There is indeed evidence that small sampling volumes from aquatic environments do not represent adequately the prokaryote composition due to microbial microscale heterogeneity (Seymour et al., 2000; Long and Azam, 2001), which supports the notion of stochastic events in small inocula. For instance, rare species responsible for keystone functions that further influence the succession of communities in the incubations may, in small inocula, stochastically cross a critical abundance threshold that allows them to proliferate during the incubation. Our observations are also in agreement with a recent study where starting communities with lower cell numbers resulted in higher variability of community succession in an artificial three species consortium (Liu et al., 2020).

Our results suggest that the less time-consuming option to directly freeze water samples (up to 10 million cells) without a preceding filtration step to concentrate cells resulted in higher CVs and similar variances. The concentration of cells on a filter further allows the complete removal of the initial water sample, avoiding the carry-over of substrates into the incubation medium and, therefore, providing additional control over the initial substrate conditions during the resuscitation process. Additionally, since the concentrated cells were cryopreserved in a liquid volume of only 2 mL, the amount of DMSO needed as a cryoprotectant is reduced to a minimum. This could be important since the ability to metabolize DMSO to dimethyl sulfide is widespread among microorganisms (Griebler and Slezak, 2000), and may bias the microbial growth of the preserved inocula after resuscitation toward species that can use DMSO as substrate. The concentration of cells via filtration before cryopreservation seems to be of particular interest for studies where carry-over of substrates from the initial sample water is undesirable or if a possible bias toward the growth of species that use DMSO as a substrate should be minimized.

Overall, the CV observed among triplicates for larger inocula in our experiments (CV = 3–35% excluding one outlier from the F-SEA incubation; Supplementary Table S4) was similar to values reported in a recently published work with triplicate incubations under controlled conditions using original, not cryopreserved natural aquatic communities as inocula (CV = 3–18%) (Morán et al., 2020; Supplementary Table S4). We therefore conclude that as long as the inocula are sufficiently large, the steps involved in the cryopreservation resulted in only limited amount of further added variation into downstream incubations than the use of natural communities as starting inocula. In this sense, increased reproducibility of starting communities in time-shifted experimental designs might bring some level of variability to simultaneously incubated replicates. This observation is, however, not surprising since additional handling steps, such as the steps required for cryopreservation, typically add variation to the outcome of experiments.

Experiment 2 was designed to test the extent of the diversity and community composition of the initial community that can be maintained after the resuscitation of cryopreserved inocula. We found that the inoculum community, the culture medium, and the interaction of both factors were relevant.

In incubations with SOLA communities, an oligotrophic and stable environment, a high degree of similarity of community composition relative to the initial SOLA community was only found in sterile-filtered original seawater treatment (F-SEA medium). This was evident in the analyses by individual ASVs (Figure 5A), ASVs grouped by order (Figure 5B), and Shannon diversity (Figure 5C). The added DOM supplements, even if reflecting to some degree the stoichiometry of the cellular content of microbes from the SOLA field station, seemingly did not promote the growth of many of the taxa present in the initial SOLA community. We assume that DOM prepared from the microbial community’s cellular contents could have been mainly composed of labile molecules, which is reflected in the higher C:N or C:P ratios as compared to the environmental DOM present at the SOLA station (Table 3). Therefore, these DOM amended treatments may have added a greater fraction of labile compounds than what is naturally found in the aquatic environment, where the recalcitrant fraction of the DOM dominates (Hansell, 2013). This likely promoted the growth of opportunists, such as members of the Alteromonadaceae family.

Conversely, Canet resuscitated communities, which originated from a highly variable source environment, attained in all incubations a comparably high degree of similarity of the community composition as well as diversity levels relative to the initial community. This result is further striking because Canet inocula were not incubated in sterile-filtered Canet water, but exclusively in media with DOM supplements that did not even originate from the Canet Lagoon (Figure 5). We speculate that these findings are linked to the adaptation of prokaryote communities to high habitat heterogeneity of the Canet Lagoon. These conditions may favor the selection of generalist species (Büchi and Vuilleumier, 2014) that accordingly grow well in a wide range of conditions, including our incubations with S-DOM or SW-DOM as growth substrates. In contrast, the oligotroph SOLA field station, featuring low habitat heterogeneity, may select for specialist species that grow well only under the specific conditions present in the initial sample water (F-SEA).

The overrepresentation of taxa in the incubations such as ASVs affiliating with the genera Alteromonas (Enterobacterales) and Arcobacter (Campylobacterales) likely reflect their niche adaptation as opportunists within the SOLA and Canet communities, respectively. Members of the genus Alteromonas are known for their ability to exploit complex organic substrates (McCarren et al., 2010) and their capability of rapid growth at high nutrient levels while outcompeting species that are abundant in natural aquatic environments (Eilers et al., 2000; Tada et al., 2011). Similarly, representatives of the genus Arcobacter were described as metabolically versatile organisms able to endure distinct habitats (Campbell et al., 2006; Snelling et al., 2006).

In contrast, Pelagibacterales, the SAR86 clade, and Puniceispirillales, which were abundant in the SOLA initial community, were generally underrepresented in the resuscitated communities, particularly those grown in ASW with DOM supplements. This is not surprising as representatives of the order Pelagibacterales are notoriously hard to grow under laboratory conditions (e.g., Sharma et al., 2013; Shen et al., 2018b). Nevertheless, members of the Pelagibacterales were actively growing, particularly in the F-SEA medium, as supported by higher cell abundances during the final incubation day compared to the incubation start (Figure 6C; positive log2-fold changes, *p*adj < 0.1). This observation is in concordance with the cultivability of the oligotrophic SAR11 clade in low nutrient sterile-seawater (Rappé et al., 2002). Similarly, members of the ubiquitous but so far uncultured marine SAR86 clade, which has streamlined metabolism (Dupont et al., 2012; Swan et al., 2013), were actively growing even if still underrepresented in F-SEA incubations. Finally, the limited growth of Puniceispirillales, that have been described as photoheterotrophs (Oh et al., 2010), may be the consequence of our incubations setup in dark conditions.

Overall, our analyses suggested that a high proportion of abundant ASVs in the initial SOLA and Canet samples was viable (Figure 6 and Supplementary Table S3). Our estimation for viability was based on the statistically tested growth of individual ASVs in triplicate incubations. In some cases, the variance of ASV abundances among the tested triplicates was large, but the growth of an ASV in a single replicate would have been sufficient to prove its viability. However, we did not have technical replicates to statistically test for the growth of ASVs in each incubation, which likely resulted in an underestimation of statistical significance used as an indicator for viability. We additionally may have underestimated the number of viable cells because we analyzed community composition based on a single data point at the end of short-term experiments, while growth for certain ASVs may have been detected only during earlier time points or during an extended incubation. Moreover, besides using filtered seawater as growth medium in one of the treatments, we did not provide growth conditions that fully reflected those present at the sample site, e.g., we incubated in dark and excluded particle attached cells via the prefiltration step in the starting communities, where the latter may have removed species interactions that were essential for some of the remaining community members. ASVs for which no growth was detected could therefore still have been viable after the cryopreservation and resuscitation procedure. Therefore, we consider the reported numbers of viable ASVs as a minimum estimate of ASVs that were effectively viable after cryopreservation.

The analyses of experiment 3 did not verify an impact of handling time during the preparation of aliquots for cryopreservation on the composition of resuscitated communities at an alpha error cut-off of 0.05. Still, our results cannot exclude that handling time may potentially have impacted the succession in community composition during the incubations: even though not significant, a trend of clustering of samples in dependency of the handling time was visible (Figures 7A,B). We therefore suggest reducing the handling time to a minimum when preparing community aliquots for cryopreservation. The handling time may influence the downstream incubations because during this period specific community members may change their activity levels compared to *in situ* conditions or even start to proliferate. It indeed had been observed that the transcriptional activities of marine prokaryotes changed differentially for different taxa at the scale of hours after sampling marine ecosystems while maintaining *in situ* conditions such as temperature and oxygen levels (Stewart et al., 2012).

Our data further indicated that long-term storage did not significantly impact community composition after the resuscitation process (Figure 7D). It needs to be pointed out that in experiment 4, differently of the other experiments, the compared incubations could not be performed simultaneously. Incubation conditions, particularly temperature, may have changed slightly since we did not use an incubation chamber for our incubations, but instead an air-conditioning system to control the temperature in the laboratory, which is less effective in maintaining exact temperatures. We believe that the observed differences in the duration of the lag-phases, as well as the time to reach the plateau (Figure 7C), may be rather a consequence of small differences in the incubation conditions than due to gradually increasing damage of cells during storage. This assumption is supported by the fact that the lag time did not successively increase with storage time, but displayed a maximum for samples with the shortest storage time, decreased to a minimum at 6 months storage, and subsequently increased again for samples stored for 12 months. In agreement with this, it has been shown previously that the incubation temperature can affect the shape of prokaryotic growth curves (Mellefont and Ross, 2003; Morán et al., 2020).

The potential differences in community composition after 5 days incubation observed between samples stored for 1.5 months and samples stored for 6 or 12 months could also be a consequence that the samples with the shortest storage time were sampled before the maximum cell density was reached, while the two latter were sampled during the plateau phase. Based on these results, we highlight the importance to ensure absolute identical incubation conditions to improve the reproducibility of cryopreserved prokaryote communities.

While we did not test storage times exceeding 12 months, it has, however, been reported that the storage of cryopreserved cells at −80°C does not fully prevent the gradual formation of ice crystals that could decrease the viability of cells after extended long-term storage times (Brockbank et al., 1992). Therefore, it seems reasonable to assume that after extended long-term storage (>12 months) at −80°C, a gradually increasing number of cells in the inoculum will be harmed, resulting in consequences for the community composition and reproducibility of incubations after resuscitation. This can be avoided by long-term storage of the cryopreserved communities at −196°C, in liquid nitrogen. Under these conditions, the cells are more stable by maintaining a vitrified state, and no damage caused by ice crystals can occur (Benson, 2008). For instance, a full recovery of the physiological capabilities of microalgae was verified after extended storage times at −196°C (Kapoore et al., 2019).

## Conclusion

This study provides evidence of a feasible method for the cryopreservation and resuscitation of natural aquatic prokaryote assemblages. Several aspects of the cryopreservation procedure (inoculum size, experimental procedure time, and storage time) were critically assessed to offer new opportunities for future research in microbial ecology based on the manipulation of natural communities. Resuscitation was achieved for contrasting communities harvested from the coastal waters of the Northwest Mediterranean Sea. Based on the findings of this study, we recommend using sufficiently large community aliquots for cryopreservation to improve reproducibility, while on the other hand avoiding extended handling time. Finally, also long-term storage times from 6 up to 12 months did not seem to cause major changes in the community composition of the resuscitated communities.

The cryopreservation protocol presented in this study can be applied to current challenges in microbial ecology since it enables to control the starting community in various experimental setups based on complex natural communities. Our method will, for instance, allow all kinds of experimental setups, where communities are mixed after a time delay and possibly in different ratios to address the priority effect after community coalescence events. Experiments could also be repeated retrospectively after the evaluation of a first experimental series, with the option to change a specific parameter in the setup while controlling for the starting community. This, for instance, could be applied to consecutively scrutinize culturing settings that cause initially rare opportunistic species to overgrow other members of their communities, with the aim to develop protocols for improved culturing of natural communities under laboratory conditions. The opportunity to completely remove the original sample medium makes the presented method furthermore highly feasible for transplant experiments, where the carry-over of substances in the original sample water should be avoided. The implementation of cryopreservation to natural communities in combination with high-throughput sequencing may furthermore increase the success for the cultivation of target organisms: preceding analyses of meta-“omic” data can be used to identify samples, where a certain target taxon is abundant and may, after the application of genome binning approaches, additionally reveal genes of the target taxon that indicate specific growth requirements. Cryopreserved communities from selected samples can then in retrospect be resuscitated under optimized conditions to enrich or even isolate the target taxon.

Overall, we believe that cryopreservation techniques applied to natural microbial communities have a high potential to advance the research field of microbial ecology. The presented tests and analyses can guide researches on the methodological aspects that should be considered for the cryopreservation of natural aquatic communities in dependency of the research question to be addressed.

## Data Availability Statement

The datasets presented in this study can be found in online repositories. The names of the repository/repositories and accession number(s) can be found below: https://www.ebi.ac.uk/ena, PRJEB38947.

## Author Contributions

SB and GPM designed the study. SB, GPM, and AR-F collected samples, performed the experiments, and analyzed the data. AR-F and SB mainly wrote the manuscript, while GPM also contributed. All authors commented on the manuscript, and read and approved the final version of the manuscript.

## Conflict of Interest

The authors declare that the research was conducted in the absence of any commercial or financial relationships that could be construed as a potential conflict of interest.

## Footnotes

1 https://benjjneb.github.io/dada2/tutorial.html

## Supplementary Material

Supplementary Figure 1 Growth of resuscitated bacterial and heterotrophic nanoflagellates (HNF) in cryopreserved not prefiltered communities from the SOLA station. Error bars represent the standard deviation among replicates (*n* = 3). Filled circles represent the mean of triplicate incubations, while empty circles illustrate individual replicates. Unfiltered surface seawater (1 L) from the SOLA station (sample day: 07/10/2020) was passed in triplicates through a 0.22 μm filter (hydrophilic PVDF, 47 mm filter, Millipore, MA, United States) using a peristaltic pump (60 mL min^–1^) to concentrate the cells. Filters were carefully transferred and completely immersed into the previously prepared 2 mL tubes with 1 mL cryoprotectant containing sterile-filtered DMSO (ACS reagent, =99.9%, Sigma–Aldrich, St. Louis, MO, United States) in 0.22 μm filter seawater. The tubes were kept at 4°C for 15 min for equilibration and then flash-frozen in liquid nitrogen and transferred to −80°C. Resuscitation of cryopreserved communities was done as described in Section “Materials and Methods.” Briefly, the cells were incubated for 8 days in triplicate incubations at *in situ* temperature (∼18°C) in 900 mL 0.22 μm filtered seawater and in dark. Daily samples were fixed with glutaraldehyde (0.1% final concentration) and stored at −80°C until flow cytometry analyses. The abundance of prokaryotes was quantified as described in the main manuscript text. The abundance of HNF cells was analyzed using the cytometer FACSCanto II (BD Biosciences, Franklin Lakes, NJ, United States) as described elsewhere (Christaki et al., 2011).

Supplementary Table 1 Summary sample processing of 16S rRNA gene amplicon sequences via the DADA2 pipeline (experiment 2).

Supplementary Table 2 GTBD taxonomic assignation and average proportion per treatment for each ASV (experiment 2).

Supplementary Table 3 L2FC and corresponding *P*-values indicating cell growth (viability) for each initially abundant ASV (>0.5% relative abundance) in the treatment with highest evidence for growth.

Supplementary Table 4 Coefficients of variation of maximal cell density for all incubations as well as for incubations incubation reported by Morán et al. (2020).

Supplementary Text 1 Step-by-step protocol for cryopreservation and the resuscitation procedure.
