# Supplementary material for: Cryopreservation and Resuscitation of Natural Aquatic Prokaryotic Communities
Source: Front Microbiol. 2021 Jan 28;11:597653. doi: 10.3389/fmicb.2020.597653 (PMC7877341; doi:10.3389/fmicb.2020.597653)
Supplement: Supplementary Figure 1 — Growth of resuscitated bacterial and heterotrophic nanoflagellates (HNF) in cryopreserved not prefiltered communities from the SOLA station. Error bars represent the standard deviation among replicates (n = 3). Filled circles represent the mean of triplicate incubations, while empty circles illustrate individual replicates. Unfiltered surface seawater (1 L) from the SOLA station (sample day: 07/10/2020) was passed in triplicates through a 0.22 μm filter (hydrophilic PVDF, 47 mm filter, Millipore, MA, United States) using a peristaltic pump (60 mL min–1) to concentrate the cells. Filters were carefully transferred and completely immersed into the previously prepared 2 mL tubes with 1 mL cryoprotectant containing sterile-filtered DMSO (ACS reagent, =99.9%, Sigma–Aldrich, St. Louis, MO, United States) in 0.22 μm filter seawater. The tubes were kept at 4°C for 15 min for equilibration and then flash-frozen in liquid nitrogen and transferred to −80°C. Resuscitation of cryopreserved communities was done as described in Section “Materials and Methods.” Briefly, the cells were incubated for 8 days in triplicate incubations at in situ temperature (∼18°C) in 900 mL 0.22 μm filtered seawater and in dark. Daily samples were fixed with glutaraldehyde (0.1% final concentration) and stored at −80°C until flow cytometry analyses. The abundance of prokaryotes was quantified as described in the main manuscript text. The abundance of HNF cells was analyzed using the cytometer FACSCanto II (BD Biosciences, Franklin Lakes, NJ, United States) as described elsewhere (Christaki et al., 2011). [file Data_Sheet_2.docx]

**Supplementary Figures**


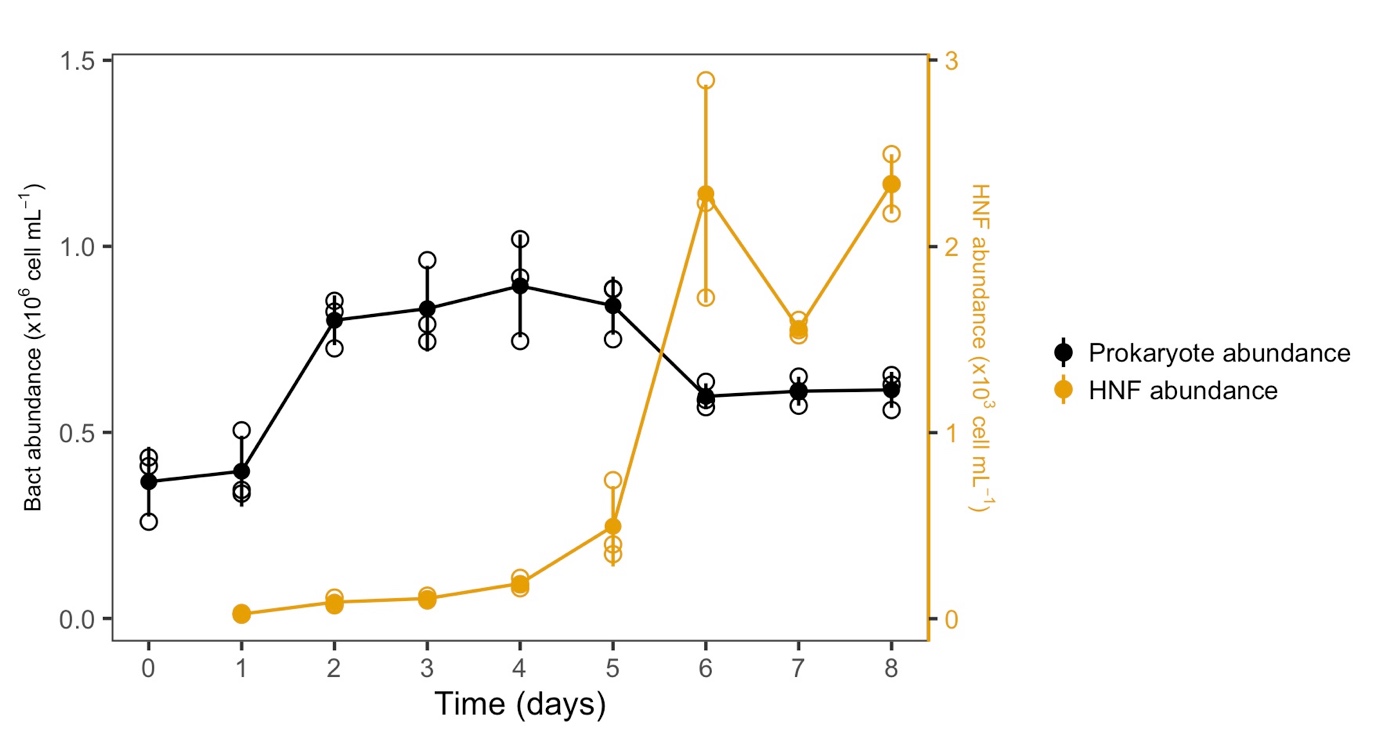


**Figure S1. Growth of resuscitated bacterial and heterotrophic nanoflagellates (HNF) in cryopreserved not prefiltered communities from the SOLA station.** Error bars represent the standard deviation among replicates (n=3). Filled circles represent the mean of triplicate incubations, while empty circles illustrate individual replicates.

Unfiltered surface seawater (1 L) from the SOLA station (sample day: 07/10/2020 ) was passed in triplicates through a 0.22 µm filter (hydrophilic PVDF, 47 mm filter, Millipore, Massachusetts, USA) using a peristaltic pump (60 mL min^-1^) to concentrate the cells. Filters were carefully transferred and completely immersed into the previously prepared 2 mL tubes with 1 mL cryoprotectant containing sterile-filtered DMSO (ACS reagent, ≥99.9%, Sigma-Aldrich, Missouri, USA) in 0.22 µm filter seawater. The tubes were kept at 4°C for 15 min for equilibration and then flash-frozen in liquid nitrogen and transferred to -80°C. Resuscitation of cryopreserved communities was done as described in the main method section. Briefly, the cells were incubated for 8 days in triplicate incubations at *in situ* temperature (~18°C) in 900 mL 0.22 µm-filtered seawater and in dark. Daily samples were fixed with glutaraldehyde (0.1% final concentration) and stored at -80°C until flow cytometry analyses. The abundance of prokaryotes was quantified as described in the main manuscript text. The abundance of HNF cells was analyzed using the cytometer FACSCanto II (BD Biosciences, New Jersey, USA) as described elsewhere (Christaki et al., 2011).

**References**

Christaki, U., Courties, C., Massana, R., Catala, P., Lebaron, P., Gasol, J. M., et al. (2011). Optimized routine flow cytometric enumeration of heterotrophic flagellates using SYBR Green I. *Limnology and Oceanography: Methods* 9, 329–339. doi: 10.1146/annurev-marine-010814-015934.
