# Supplementary material for: Cryopreservation and Resuscitation of Natural Aquatic Prokaryotic Communities
Source: Front Microbiol. 2021 Jan 28;11:597653. doi: 10.3389/fmicb.2020.597653 (PMC7877341; doi:10.3389/fmicb.2020.597653)
Supplement: Supplementary Text 1 — Step-by-step protocol for cryopreservation and the resuscitation procedure. [file Data_Sheet_1.DOCX]

**Supplementary Materials**

**Supplementary Text S1. Step-by-step protocol for cryopreservation and resuscitation procedure**

1. Prepare a sterile stock solution of Dimethyl sulfoxide (DMSO, ACS reagent, ≥99.9%, Sigma-Aldrich, Missouri, USA) by sterile filtration (0.22 µm filter, hydrophilic PVDF, 25 mm filter, Millipore, Massachusetts, USA).

For cryopreservation of filters proceed to step IIa, while for cryopreserved water samples proceed to step IIb.

1. Cryopreservation of concentrated inocula:
2. Prepare a DMSO working solution by adding the filtered DMSO to fresh artificial seawater (ASW) medium reflecting the salinity of the original sample (alternatively, sterile filtered seawater can be considered) to a 5% (v/v) concentration.
3. Add 1000 µL DMSO working solution into standard 2 mL tubes (one tube per filter), keep the tubes at 4°C until use.
4. Filter sample water from a volume containing ≥ 500 x10^6^ cells (after 0.8 µm prefiltration to remove protists) with a vacuum pump or a peristaltic pump through 0.22 µm filter (e.g. hydrophilic PVDF, 25 mm filter, Millipore, Massachusetts, USA) (vacuum pump: ~600 mg Hg; peristaltic pump: (~60 mL min^-1^). If using a vacuum pump carefully avoid drying of the filters, by reducing the pressure at the end of the filtration to a minimum to prevent cell damage^1^.
5. Next, immerse the filter completely into the DMSO working solution resuspended cells attached to the filter by pipetting up and down. Keep the tube 15 min at 4°C for equilibration.
6. Cryopreservation of aquatic microbial communities for sample water:
7. Prepare aliquots of 0.8 µm pre-filtered water samples without prior cell concentration via filtration.
8. Add filtered DMSO to the aliquots prepared in the preceding step (DMSO 5% final concentration).
9. Keep the aliquots for 15 min at 4°C.
10. Flash-freeze the community aliquots in liquid nitrogen, then transfer it to -80°C for long-term storage.
11. Thaw the cryopreserved samples at room temperature for about 30-45 min to initiate the resuscitation.
12. Add the whole content of the defrosted tubes (filter together with the DMSO solution and the resuspended cells; step IIa) or the frozen liquid sample aliquots (step IIb) into freshly prepared growth medium to regrow the cryopreserved communities.

^1^ This step slightly differs from the protocol performed on samples presented in the manuscript where we resuspended the cells by pipetting up and down ASW medium on the filter while it was still placed on the filter device. For later preparation of cryopreserved community aliquots, we however followed the protocol as detailed here.
